# Helping babies breathe: assessing the effectiveness of simulation-based high-frequency recurring training in a community-based setting of Pakistan

**DOI:** 10.1186/s12887-021-03014-2

**Published:** 2021-12-08

**Authors:** Kiran Mubeen, Marina Baig, Sadia Abbas, Farzana Adnan, Arusa Lakhani, Shireen Shehzad Bhamani, Bushra Rehman, Shahnaz Shahid, Rafat Jan

**Affiliations:** 1 Aga Khan University School of Nursing and Midwifery (AKUSONAM), Karachi, Pakistan; 2 Integrated Reproductive Maternal, Newborn, Child Health and Nutrition program, Punjab (IRMNCH), Lahore, Pakistan

**Keywords:** Community midwives, Newborn resuscitation, Pakistan, Helping baby breathe, Low dose high frequency, Simulation

## Abstract

**Background:**

Birth asphyxia is one of the significant causes of neonatal deaths in Pakistan. Poor newborn resuscitation skills of birth attendants are a major cause of neonatal mortality in low resource settings across the globe. This study aimed to evaluate the effectiveness of the Simulation-Based High-Frequency training of the Helping Babies Breathe for Community Midwives (CMW), in district Gujrat, Pakistan.

**Method:**

A pre-post-test interventional study design was used. The universal sampling technique was employed to recruit 50 deployed CMWs in the entire district of Gujrat. The pre-tested module and tools of Helping Babies Breathe (2nd edition) were used in the intervention. Using the High Frequency training approach, three one-day training sessions were conducted for CMWs at an interval of 2 months. During the 2 months interval, participants were monitored and supported to practice their skills at their birthing centers. Knowledge and skills were assessed before and after each session. The McNemar and Cochran’s Q tests were applied for data analysis. Participants’ feedback was also obtained at the end of each training, which was analyzed through descriptive statistics.

**Results:**

Data from 34 CMWs were analyzed as they completed all three training sessions and assessments. The results were statistically different after each training session for OSCE B (*p*-value < 0.05). However, for knowledge and OSCE A, significant improvement was observed after training sessions 1 and 2 only. Pairwise comparison showed that pre-assessment at training 1 was significantly different from most of the repeated measures of knowledge, OSCE A, and OSCE B. Moreover, the learners appreciated the overall training in terms of organization, content, material, assessment, and overall competency. Additionally, due to a small sample size of the CMWs, and a short time of the intervention, significant differences in morbidity and mortality outcomes could not be detected.

**Conclusion:**

The study concluded that a series of training and continuous supportive supervision and facilitation enhances Helping Babies Breathe (HBB) knowledge retention and skills. The study recommends, periodic, structured and precise HBB trainings, with ongoing quality monitoring activities through blended learning modalities would help sustain and scale-up the intervention.

## Background

In 2015, 2.7 million newborns died globally, out of which 0·691 million deaths occurred due to intrapartum-related events [1]. Two-thirds of the world’s neonatal deaths occurred in just ten countries, mostly in Asia [2]. South Asia has a neonatal mortality rate of 43 per 1000 live births and a still birth rate of 35 per 1000 live births [3]. Around, 40-45% of these deaths in South Asia occurred during labour, delivery, and the first 24 h of birth [3].

Pakistan occupies the first position among these countries [4] with a neonatal mortality rate of 42 deaths per 1000 live births [5]. One of the major reasons for neonatal mortality is suggested to be the occurrence of 48% of the births outside health facilities [5] and lack of proper resuscitative facilities [6]. The United Nations International Children’s Education Fund (UNICEF) report [7] shows that, in Pakistan, 39.3% of neonatal deaths are caused by prematurity, 20.9% by birth asphyxia, and 17.2% by sepsis. The last three demographic health surveys of Pakistan indicates a 14% reduction in neonatal mortality rates [5]; however, the proportion of newborn deaths occurring within the first 24 h of birth has remained high (i.e. 36.2% of all neonatal deaths) [8]. These grave figures strongly suggest that with a similar pace of progress, Pakistan will lag in achieving the Sustainable Development Goals (SDGs) related to reducing neonatal mortality [9].

Execution of effective community-based interventions can prevent two-thirds of the mortalities [10]. Helping Babies Breath (HBB) is a low-cost, simplified form of the Neonatal Resuscitation Program (NRP) [11], that specifically focuses on the implementation of basic resuscitation skills. These skills are most often sufficient to support the health of 99% of newborns in a way that is specifically oriented for low-resource settings, including the didactic and clinical equipment that is included in the program materials [12].

Traditional capacity-building approaches, which include extended, off-site, group-based workshops, alone are found to be ineffective for long-term impacts on the knowledge and skills retention of the service providers and optimizing sustained improvements in service delivery [12–16]. Conversely, “Low Dose High Frequency (LDHF) capacity-building approaches promote maximal retention of clinical knowledge, skills, and attitudes, through short, targeted in-service simulation-based learning activities, spaced over time, and reinforced with structured, ongoing practice sessions on the job” [17]. Several studies from low and middle-income countries, including Nepal, India, Kenya, Tanzania, Sudan, and Rwanda, showed that HBB training for birth attendants, with frequent refreshers and on job practice, positively impact on the retention of knowledge and skills, and yield subsequent improvement in neonatal mortality outcomes [16, 18–20].

It has been projected that approximately 80% of newborn deaths are preventable by having skilled birth attendants, especially midwives who work in communities and are the pillars of the primary healthcare system in Pakistan [21]. A cross-sectional survey assessed the knowledge of Lady Health Visitors (LHVs) and midwives, working at primary healthcare facilities regarding neonatal resuscitation. The study found a need for regular practical in-service training of LHVs and midwives regarding basic neonatal resuscitation; and recommended that such training should be made a pre-requisite for getting a job [6].

In Pakistan, a two-year Community Midwifery (CMW) program has been in place since 2007, which aims to train CMWs to serve their communities and run independent birthing centers. In terms of neonatal survival at birth, the CMW curriculum includes a neonatal resuscitation program, but has limitations regarding its application in low resource rural settings, due to the absence of intensive care services, inadequate periodic competency-based training, and lack of trained staff in referral facilities [11]. Therefore, it was imperative to identify community-based, low-cost interventions, to reduce neonatal deaths related to birth asphyxia [22, 23]. Although a study conducted in low resource settings in Pakistan assessed the effectiveness of the HBB training [15]; the effectiveness of the training approach in the unique context of Community Midwives needed further exploration. In the Pakistani context where community midwives operate at distant locations, applying the LDHF Training approach in the community-based setting was challenging and highly resource-intensive. For applying LDHF in remote settings, CMWs had to perform consistent, scheduled practice and emergency drills over a sufficient time under the supervision of their mentors to enable consolidation of skills [17], which was highly ambitious. Therefore, it was important to assess a modality that is cost-effective as well as doable in the community midwifery context. Considering the above challenges, this study aimed to evaluate the effectiveness of the simulation-based high-frequency recurring training of the Helping Babies Breathe for Community Midwives (CMW), in district Gujrat, Pakistan. The objectives of the study were to:

- Evaluate the effectiveness of simulation-based high-frequency trainings of the Helping Babies Breathe program on the knowledge and skills retention of Community Midwives
- Assess the participants’ satisfaction with the training approach
- Determine the effect of the intervention on newborn morbidity and mortality indicators

## Methods

### Study design

A pre-post interventional design was used to achieve the research objectives.

### Study population and setting

The HBB training intervention was implemented for the CMWs of district Gujrat which is situated in the northern part of Punjab (the largest province of Pakistan), and has a population of 2.756 million. CMWs work as private practitioners and report to the Integrated Reproductive Maternal, Newborn, and Child Health and Nutrition (IRMNCH) Program, which is the provincial regulatory body of CMWs.

### Participants recruitment

After receiving their written permission, the IRMNCH Program Head was contacted and requested to share a list of the CMWs deployed in district Gujrat. At the time of the intervention, a total of 50 CMWs and three CMW tutors were deployed in district Gujrat, and all were reporting to the IRMNCH office. Therefore, the universal sampling technique was applied. Based upon the research team’s request, the IRMNCH program head approached all the CMWs meeting the eligibility criteria, and their midwifery tutors, to participate in the training. The purpose of the study was explained to the participants before the first large group session. Based on their willingness, written informed consent was obtained.

### Eligibility criteria

- Licensed by the PNC
- Deployed in Gujrat
- Reporting to the IRMNCH program

### Training site

In Pakistan, CMWs function independently in their clinics, which are distantly situated. To train them using the simulation-based high-frequency recurring training modality, the research team organized sessions at a central location. The training site and logistics were arranged by the IRMNCH office. The CMWs were required to travel three times to the training location, which was in the same district in which they were located. Therefore, the IRMNCH office recommended using Aziz Bhatti Shaheed Hospital, Gujrat, the same institution from where CMWs had received their basic Community Midwifery education for all three large group training sessions.

### Data collection tools

‘The HBB knowledge check’ [24], Objective Structured Clinical Evaluation (OSCE), and Bag-Mask Ventilation (BMV) [25] checklists were adopted from the American Academy of Pediatrics’ HBB Toolkit (2nd Edition) to assess the knowledge and skills of the midwives during the intervention. These tools have been validated [26] and used in other similar settings [15, 22], where HBB capacity building interventions have been implemented. The knowledge check tool consists of 18 multiple- choice questions (MCQs); 80% of correct responses were considered a ‘Pass’. The research adopted a similar cut-off to the one used for the HBB knowledge check (1st edition) [15] because, at the time of the intervention, the knowledge cut off for the HBB tool (2nd edition) was not available in published studies. For the HBB skills assessment, OSCE A and OSCE B [27] were used. OSCE ‘A’ assessed the routine care and the initial interventions during the first 60 s (first 1 minute) of the newborn’s life, known as the Golden Minute. The checklist of OSCE A consists of 12 items, of which achievement of nine was considered as a ‘Pass’ [28]. The OSCE B measured the components of OSCE A, BMV tool, and decision making based on the heart rate to initiate ventilation. The OSCE B tool consists of 23 items, in which a score of 17 was considered a ‘Pass’ [28]. The study monitoring aspect utilized the BMV checklist, in which the component of infection prevention practices was added.

The training evaluation tool was adapted from the American Academy of Pediatrics [29]. It consisted of participants’ feedback on the training organization, content, material, assessment, and overall competence. A Likert scale of 1 (Strongly Disagree) to 5 (Strongly Agree) was used to assess each domain.

For assessing the effectiveness of the training on newborn morbidity and mortality indicators, the research team also consulted the IRMNCH program periodically, which collected monthly reports from the CMWs, entered them in the CMWs’ Management Information System (MIS). The monthly reports were analyzed during the period of study, from June 2017 to March 2018, to detect differences in newborn morbidity and mortality indicators. During the study period, the following relevant indicators were available in the IRMNCH program’s CMW MIS for analysis in regards to achieving the third objective of the study.

- Number of deliveries
- Number of live births
- Number of newborns with asphyxia
- Number of newborns referred due to asphyxia
- Number of still births

### Phases of data collection

The data collection was conducted during the three phases of the study: 1) Pre- intervention; 2) Intervention; and 3) Post-intervention. Table 1 shows the timeline of each phase and the details are as follows:

**Table 1:** Timeline of research phases

Phase I	Phase II	Phase III
May-August, 2017	September-January, 2018	February-March, 2018

### Phase I - pre-intervention phase

#### Collection of baseline data

The baseline data of newborn morbidity and mortality indicators from May-August, 2017 was obtained from the IRMNCH office.

#### Training of trainers

At the time of the study, only three community midwifery tutors were deployed in district Gujrat. They were registered nurse-midwives who had clinical and teaching experience of more than 15 years. These tutors denied having received any formal training in HBB; however, they were aware of the basic concepts of newborn resuscitation. These three midwifery tutors agreed to become the trainers of HBB, and to conduct CMWs’ technical supervision in their work settings.

A one-day training of trainers was conducted by the Primary Investigator (PI) and two other members of the research team. The three CMW tutors and the three members of the research team made a group of six facilitators who conducted the trickle-down CMW trainings. This group remained consistent throughout the intervention phase, including monitoring and evaluation. The training content for trainers included the HBB theory and skills (2nd Edition), contemporary teaching-learning methodologies, and roles and responsibilities as a co-trainer and field supervisor [29]. A pre- and post-test of trainers were conducted, using the standard tools of the American Academy of Pediatrics, and it was ensured that the trainers successfully completed the HBB training. After the trainers were prepared, they were expected to assist the research team in the implementation of the simulation-based high-frequency training and in the monitoring of the research, by submitting a monthly monitoring checklist to the research team. The trainers (Community Midwifery Tutors) were able to maintain their competencies by participating in the CMWs’ training along with the trainers from the research team as well as to conduct knowledge and skill assessment.

### Phase II – intervention/implementation phase

#### Trickle down training using the high-frequency approach

The implementation phase was spread over 5 months, from September, 2017 to January 2018, during which three training sessions were conducted for the CMWs. The first training was conducted in September, 2017, the second in November 2017, and the third in January, 2018, with 43, 42, and 39 CMWs, respectively. Considering that the CMWs would not be able to commute frequently for skills practice, it was decided to deliver all the content in 1 day, followed by group practice and skills sign-off.

**Fig. 1:**
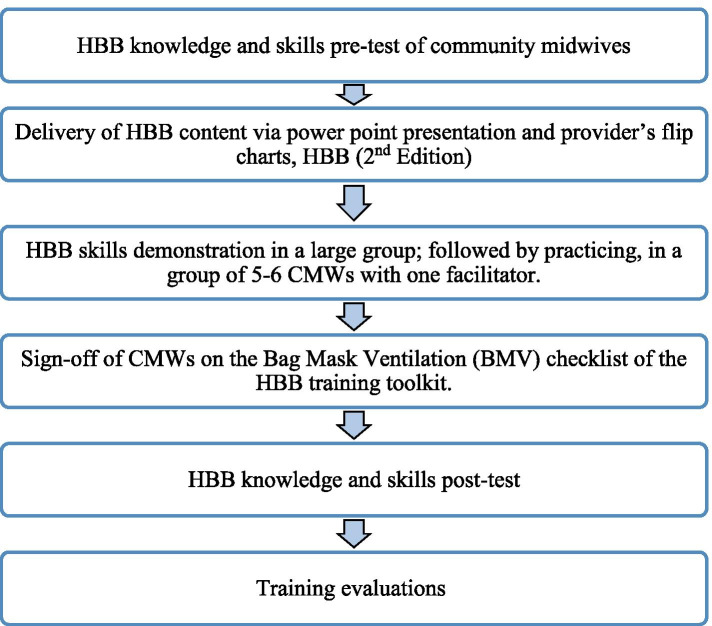
Community Midwives’ Training Process

After one initial training, two refreshers were provided to the CMWs, at an interval of 2 months each. Pre and post-tests of knowledge and skills were conducted in every training. Since, accommodating all the CMWs (n = 43) in a day was not possible, the group was divided into two for the training. For both groups, the initial workshop was comprised of 8 hours (1 day) of training, followed by two refreshers of six and 5 hours respectively including time for pre- and post-tests, training evaluations and breaks. During the refresher training sessions, the key areas of the course were reviewed, including areas needing improvement identified during the skills re-demonstration, followed by an in-depth question answer session. In each training session, the activities were undertaken are shown in Fig. 1:

#### Quality checks and compliance

After every training session, ongoing monitoring of the intervention, as well as technical supervision through visits to their birthing clinics, was continued by the trainers. The supervision began in September 2017, is, soon after the first training, and ended in March, 2018.

During the implementation phase, trained supervisors randomly visited 10% of the participants every month, to assess the application of the HBB skills in the CMWs’ work settings. The visits were scheduled between training sessions. The trainers were provided with a standard monitoring checklist, which they were required to submit to the research team. During the intervals of each training, these facilitators paid unscheduled visits to the participants’ birthing centers, along with the Neonatalie Simulator. The CMWs were given scenarios and they were asked to demonstrate their HBB skills. The trainers also assessed the newborn resuscitation set-up and infection prevention practices in their birthing centers.

### Phase III – post-intervention phase

This phase was based on the analysis of outcomes available in the CMW MIS of IRMNCH, pre and post-tests, and course evaluations. A comparison was made between the baseline, midline, and endline results of pre and posttests. The training evaluation and participant feedback were obtained through both qualitative and quantitative methods. A qualitative evaluation of training effectiveness was also conducted by a neutral evaluator, who was not a part of the research team, 3 months after the third training. The findings of this evaluation will be published elsewhere [30]. In this paper, the quantitative assessment of the training evaluation is presented.

### Statistical analysis

Analysis was performed using the IBM SPSS Statistics version 20. Descriptive statistics were computed for categorical variables by computing their frequencies and percentages. Quantitative variables were computed as mean and standard deviations. The McNemar test was conducted to compare the dichotomous dependent variable (Pass/ Fail) for Knowledge, OSCE A, and OSCE B, before and after the HBB training, at baseline, midline, and endline, individually. Moreover, we used Cochran’s Q test to determine whether the proportion of CMWs who had passed knowledge, OSCE A and OSCE B, was different between all the repeated measures. A *p*-value of < 0.05 was considered significant throughout the study.

## Results

### Participants

**Fig. 2:**
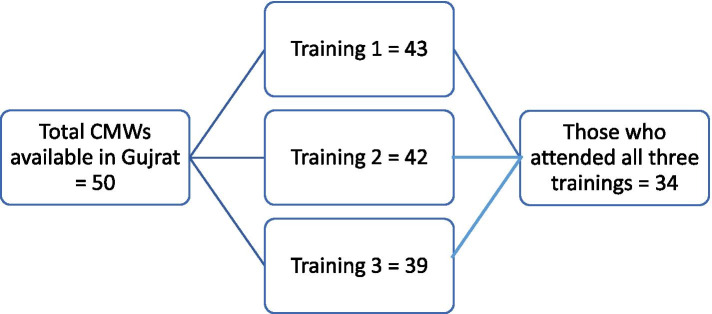
Number of participants in each training

Forty-three CMWs were enrolled in the first training; however, a total of 34 CMWs attended all three consecutive trainings (Fig. 2). The data of these 34 CMWs were included for the analysis of pre and post test results.

The mean age of all the participants was 26.12 years (SD 3.36), with a range of 22 to 35. The majority of the study participants had received education up to Grade 10 level (71%), 26% were educated at Grade 12 level and only 3% had Bachelor’s or Master’s degrees. The mean years of experience of all the participants were 2.33 years (SD 1.63), with a range of 6 months to 10 years.

### Effect of intervention on the knowledge and skills retention of community midwives

Table 2 shows the results of McNemar’s and Cochran’s Q-test. An exact McNemar’s test determined that there was a statistically significant difference in the proportion of CMWs who passed, pre and post-intervention. The results were statistically different after each training for OSCE B (*p*-value < 0.05); specifically, after training one, the number of CMWs who passed increased from 6 to 26; after training two, the number of CMWs who passed increased from 20 to 29; and a significant increase was found after the third training pre- (*n* = 23) to post-test (*n* = 32). This trend highlights that there was a significant improvement in the passing proportion after each training. However, for knowledge and OSCE A significant improvement was observed after training one and training two only. After training one and two, the knowledge of CMWs showed significant improvement, as evident in the increased passing numbers rising from pre (*n* = 17) to post (*n* = 23), and pre (*n* = 22) to post (*n* = 30), respectively. A similar outcome was found for OSCE A after training one and two when the number of CMWs who passed increased between pre (n = 3) to post (*n* = 29), and after training two, from pre (*n* = 24) to post (*n* = 34). No change was observed after training 3 in knowledge and OSCE A, which suggests that the CMWs were retaining the knowledge and performing the skills of OSCE A at a satisfactory level.

**Table 2:** HBB training effect on the passing scores of CMWs’ knowledge and OSCE (A & B)

Trainings	Assessments	Knowledge		***p*** values	OSCE A		***p*** values	OSCE B		***p*** values
	Passing Score	14 out of 18			9 out of 12			17 out of 23		
		Pre	Post		Pre	Post		Pre	Post	
		n (%)	n (%)		n (%)	n (%)		n (%)	n (%)	
Training 1	Pass	17 (50)	33 (97.1)	< 0001*	3 (8.8)	29 (85.3)	< 0001*	6 (17.6)	26 (76.4)	< 0001*
Training 2	Pass	22 (64.7)	30 (88.2)	0.008*	24 (70.5)	34 (100)	0.002*	20 (58.8)	29 (85.3)	0.012*
Training 3	Pass	31 (91.2)	33 (97.1)	0.625	31 (91)	34 (100)	0.25	23 (67.6)	32 (94)	0.022*
**Cochran Q**		41.12			103.7			61.65		
**p values**		< 0001**			< 0001**			< 0001**		

*significant at *p* value < 0.05 by Mc Nemar’s Test

**significant at *p* value < 0.05 by Cochran’s Q test

Table 2 also shows the outcomes of Cochran’s Q test, which indicated that there was a significant difference among all six measures of knowledge, OSCE A, and OSCE B. Pairwise comparison showed that the results of pre-assessment at training one were significantly different from most of the repeated measures of knowledge, OSCE A, and OSCE B. This finding highlights the need for HBB training for CMWs. However, pairwise insignificant differences were found for pre-assessment at training 2 and 3, which indicates the need for repeated training to sustain the CMWs’ knowledge and skills over time. Hence, it is evident that the overall results of the training were effective in improving the scores of knowledge and OSCE.

### Participants’ satisfaction with the training approach

The training evaluation tool consisted of participants’ feedback on the training organization, content, material, assessment, and overall competence. The range of each domain varies as per the items; specifically, Organization (4 to 20), Content (2 to 10), Material (9 to 45), Assessment (3 to 15), and Overall Competency (1 to 5).

**Fig. 3:**
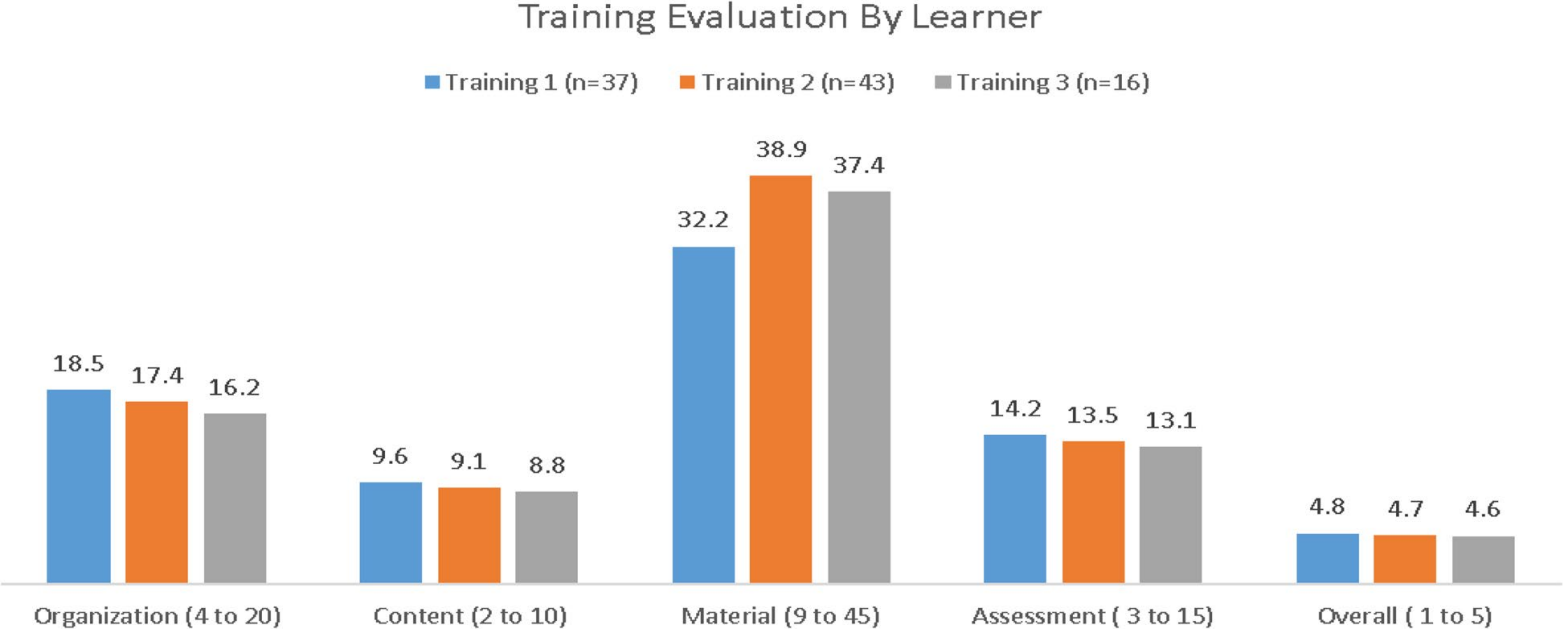
Training Evaluation of all three trainings

Figure 3 refers to the mean scores of each domain, as evaluated by the learners after each training. The higher the mean, the higher the agreement and/or level of satisfaction. As evident in the graph, each domain was rated positively, which indicates that the learners appreciated the overall training in terms of organization, content, material, assessment, and the overall competency gained during each training. However, slightly declining trends were observed in the means scores from the first training to the third training, in all the domains, except materials. The learners found that the course materials were more helpful in training two and three than in the first training.

The analysis of the comments section showed repeated praise for the facilitators’ knowledge and competence, which helped the participants in meeting the course objectives. Participants also appreciated the various teaching-learning strategies utilized during the trainings. The participants found supervised practice in groups and self-check questions very beneficial. They shared that the variety of strategies increased their learning and stimulated their intellectual curiosity. The course material utilized by the facilitators was reported as highly effective, in all the training evaluations.

Besides the many positive points, some of the participants also shared their limitations in attending all three training sessions. They expressed difficulties, such as bad weather and long travel, which resulted in absence from one or more workshops. Moreover, the participants requested the time duration of the training should be increased; they suggested that each training be delivered in two half-days instead of a long 6-8 h session. Overall, the participants expressed satisfaction and thanked the team for giving them the opportunity to learn an essential lifesaving skill.

### Effect of the intervention on newborn morbidity and mortality indicators

Table 3 shows an increase in the number of asphyxia, 3.6% (*n* = 23/632 live births) in baseline, to 4.04% (*n* = 14/346 live births) in the midline, and 6.6% (n-17/255 live births) towards the end line. However, a decreasing trend was observed in the stillbirth rates from 20.5 (*n* = 13/632 live births) at baseline to 19.8 (*n* = 7/346 live births) at midline, and 19.6 (*n* = 5/255 live births) at the end line.

**Table 3:** Comparison of Outcome Indicators for all time interval

Outcomes	Baseline	Midline	End Line
	(May-Aug, 2017)	(Sept-Oct, 2017)	(Jan-Mar, 2018)
**Number of deliveries**	642	353	260
**Live births**	632	346	255
**Asphyxia**	23 (3.6)	14 (4.04)	17 (6.6)
**Referred due to asphyxia**	23	14	17
**Still birth rate/1000 Live Births**	13 (20.5)	7 (19.83)	5 (19.60)

## Discussion

The primary aim of this study was to assess the effectiveness of the simulation-based high-frequency training approach, along with low dose on-site supervision, on the knowledge and skills retention of Community Midwives, related to the lifesaving skills of Helping Babies Breathe, over 5 months. The results of the pre and post-tests of knowledge and skills of each training showed that HBB training, in a community-based setting, using the simulation-based high-frequency training approach, can bring a positive change in the participants’ practice, as evidenced by improvement in the knowledge and OSCE pre- and post-test scores.

In the current study, the lowest scores were observed in the baseline pre-test, which shows that the deployed CMWs were not well versed with the concepts of newborn resuscitation, which is an area of concern. It implies that although the CMWs attended many births, their practices lacked the evidence-based approaches presented during the first session. This implies the need for CMWs’ curriculum strengthening by introducing HBB as a basic competency for qualifying as a CMW. Our qualitative evaluation of this training [30], conducted 2 months after all the trainings, showed that the CMWs were never taught skills in the way that they were taught during the HBB training. The skills teaching format in their pre-service education was largely knowledge (content) driven and lacked sufficient practice opportunities [30]. This again is an area for faculty development in this district, to ensure that both knowledge and skills are delivered in such a manner that adequate psychomotor competency is achieved.

Another important finding is that no change in knowledge and OSCE A was observed after the second session, indicating adequate knowledge retention after continuous training; however, this was not the case for OSCE B which involves critical steps of BMV. Similar findings are reported by an earlier study that knowledge retention becomes evident earlier rather than skills [13]. Therefore, ongoing refresher training and continuous supportive supervision and facilitation on HBB are required to maintain the skills of neonatal resuscitation [23, 31, 32]. In addition, for rigorous HBB training, ongoing quality monitoring activities should also be planned and applied [33]. Hence, the overall results suggest that regular training is effective in improving the scores of knowledge and OSCE.

Due to a small sample size of the CMWs, and a short time of the intervention, significant differences in newborn morbidity and mortality outcomes could not be detected. Furthermore, since the CMWs were looking after low-risk clients only, and referred the patients with complications, including the asphyxiated newborns, therefore, data regarding neonatal deaths due to asphyxia was not available.

At the time of the intervention, a total of 50 CMWs were practicing in the district. However, participants in all the training sessions were not the same. We inducted 43 CMWs in the first training, but the 2nd training had new participants and we lost a few from the first training. During the 3rd training, we faced severe weather conditions like fog and rain, resulting in many CMWs being unable to reach the training facility. In total, we lost nine participants who could not complete all three training sessions. The reasons for dropouts were mainly non-availability of transport, being sick, or having a busy birthing center on training day. Though the reasons for missing the training were genuine, for scaling up the intervention, the possibility of work-based or online training should be explored in the future.

The factors behind the overall success and effective implementation of the training were the competent midwifery tutors of Gujrat, who carried out the testing and the monitoring activities as part of their normal duties. No master trainers were hired for the study and we were able to utilize and develop the capacities of the existing human resources in the health system. Hence, this approach ensured the sustainability of the intervention. Moreover, the robust monitoring mechanism of the IRMNCH program and their constant support helped in maintaining the checks and balances of the training and its implementation. The participants’ thirst for knowledge and their motivation to learn was encouraging, and we faced minimal attrition, considering their circumstances.

### Study limitations & recommendations

Provided that the CMWs and their mentors were scattered at distant locations, on-site low dose trainings with weekly coaching and practice was not possible in our context because it required to travel for either the mentors or the training participants. Based on the logistic concern, participants were gathered at a central location for a day-long training. However, they verbalized their concern to have trainings with a shorter duration, spread over 2 days. Applying this recommendation may lead to further drop outs as we experienced during the last training day that had the lowest number of participants. Therefore, for scale-up and sustainability, the study recommends the placement of mannequins and self-study resources in each CMWs’ practice setting with virtually proctored practice sessions by peer mentors.

Since the study was conducted in one district and on small sample size, the findings of the study cannot be generalized. However, study findings imply the development of a large scale intervention with increased sample size and in diverse study settings to strengthen the validity and applicability [34].

Previous studies applying LDHF on large samples show a reduction in early neonatal mortality rates within the first 24 h of birth and by the 7th day of life [33, 35, 36]. These studies were conducted in facilities where registries and strong MIS were available. Our setting was purely community-based, where the CMWs work as private practitioners and the MIS largely depends on CMWs’ self-reported data. Therefore, it was difficult to show the impact on the neonatal outcomes in the timeframe of 11 months, with a limited sample. As was suggested in our literature review, follow- up is challenging in low-resource settings [36]. For future and scaled-up interventions we recommend using strong monitors for data collection and inclusion of basic health units for a larger sample and a comprehensive MIS.

## Conclusion

The Helping Baby Breathe trainings with simulation-based high-frequency recurring workshop modality enhanced the knowledge and skills of community midwives in district Gujrat, as a progressive trend of improvement was observed before and after each training session. The analysis confirmed that this approach is effective in not only enhancing their knowledge and skills but also in retaining their competencies over time, including identification of signs of asphyxia and referring accordingly. The analysis of training evaluation also confirmed the delivery of quality training during each phase. Although, the results of this pilot test are encouraging, a large-scale randomized controlled trial with larger sample size, with a blended modality for weekly proctored practice under peer supervision, may prevent drop outs and help to scale-up the intervention in the unique context of Community Midwifery model in Pakistan.

## Data Availability

The datasets used and/or analysed during the current study are available from the corresponding author on reasonable request.

## Abbreviations

CMW: Community Midwives
HBB: Helping Babies Breathe
OSCE: Objective Structured Clinical Examination
LDHF: Low Dose High Frequency
UNICEF: United Nations International Children’s Education Fund
SDGs: Sustainable Development Goals
NRP: Neonatal Resuscitation Program
IRMNCH: Integrated Reproductive Maternal, Newborn, and Child Health and Nutrition
BMV: Bag-Mask Ventilation
MIS: Management Information System
